# Successful Management of a Young Patient Suffering From Pyoderma Gangrenosum Following Gastric Bypass Surgery: A Case Report

**DOI:** 10.7759/cureus.34305

**Published:** 2023-01-28

**Authors:** Abdulqader M Albeladi, Zaki Busbaih, Ali A Almohammed Saleh, Abdullah Q AlAlwan, Mohammed A Aljughayman, Almunthir S Alhamed, Abdulmohsen Alsuwaigh, Abdulmohsen Aleasa, Tayseer Alali, Mohammad S AlGhadeer, Sajedah A Albeladi

**Affiliations:** 1 General Surgery: Laparoscopy, Prince Saud Bin Jalawi Hospital, Al Ahsa, SAU; 2 Medicine, King Faisal University, Al Ahsa, SAU; 3 General Surgery: Bariatry and Laparoscopy, Prince Saud bin Jalawi Hospital, Al Ahsa, SAU; 4 General Surgery, Prince Saud bin Jalawi Hospital, Al Ahsa, SAU; 5 General Surgery, Prince Saud Bin Jalawi Hospital, Al Ahsa, SAU; 6 General Surgery, King Faisal Hospital, Al Ahsa, SAU

**Keywords:** pyoderma gangrenosum, successful outcome, biologic treatment, debridement tissue, idiopathic pyoderma gangrenosum

## Abstract

Pyoderma gangrenosum (PG) is a pathogenetically ill-defined neutrophilic dermatosis frequently characterized by severely painful ulcerations with no identifiable infective pathogens. There are no diagnostic criteria for PG, nor specific gold standard management, which may complicate the process of dealing with patients suffering from this disease. Here, we report a case of a 27-year-old male patient, with a history of gastric bypass surgery three years ago, who presented with a left leg non-healing ulcer diagnosed as a PG by the clinical presentation and biopsy of the ulcer. He was managed by the administration of systemic immunomodulators, a surgical debridement procedure, and the application of a vacuum. The patient was discharged with vitamin B complex and vitamin D supplements as well as zinc sulfate and folic acid. Also, multiple doses of Infliximab intravenously and vitamin B 12 intramuscularly result in a satisfactory healing process of the ulcer. Since PG is a diagnosis of exclusion, clinicians must be aware of the need for highly specific history-taking, previous surgical history, laboratory investigations, and histopathological workup in order to reach the diagnosis.

## Introduction

Pyoderma gangrenosum (PG) is a rare, non-infectious, immune-mediated dermatosis. Along with Sweet's disease and Behcet's illness, it falls under the umbrella of neutrophilic dermatosis [1]. Although the pathogenesis of PG is not well-understood, recent studies suggest that clonal T growth, increased levels of inflammatory mediators (interleukin (IL) 17, IL 23), and genetic susceptibility all contribute to its development [2]. Previously, it was assumed that these lesions had an infectious etiology. However, over 50% of the patients had systemic diseases such as Inflammatory bowel disease, arthritis, and hematological malignancy associated with PG [3-4]. Clinically, the hallmark of PG is a painful, sterile, necrotizing ulceration that can manifest as a single, isolated lesion or a widely diffused lesion [5].

Making a diagnosis of PG remains challenging due to the lack of definitive laboratory or histological diagnostic criteria and is therefore referred to sometimes as a diagnosis of exclusion [2]. Furthermore, treatment options for PG vary greatly, as there is still no established gold standard regimen [6]. In this article, we report a case of a 27-year-old male patient, with a previous history of gastric bypass surgery, who presented with a left leg non-healing ulcer diagnosed as PG, which was treated by the administration of systemic immunomodulators in conjunction with a surgical debridement procedure that was followed by the application of a vacuum.

## Case presentation

**Figure 1:**
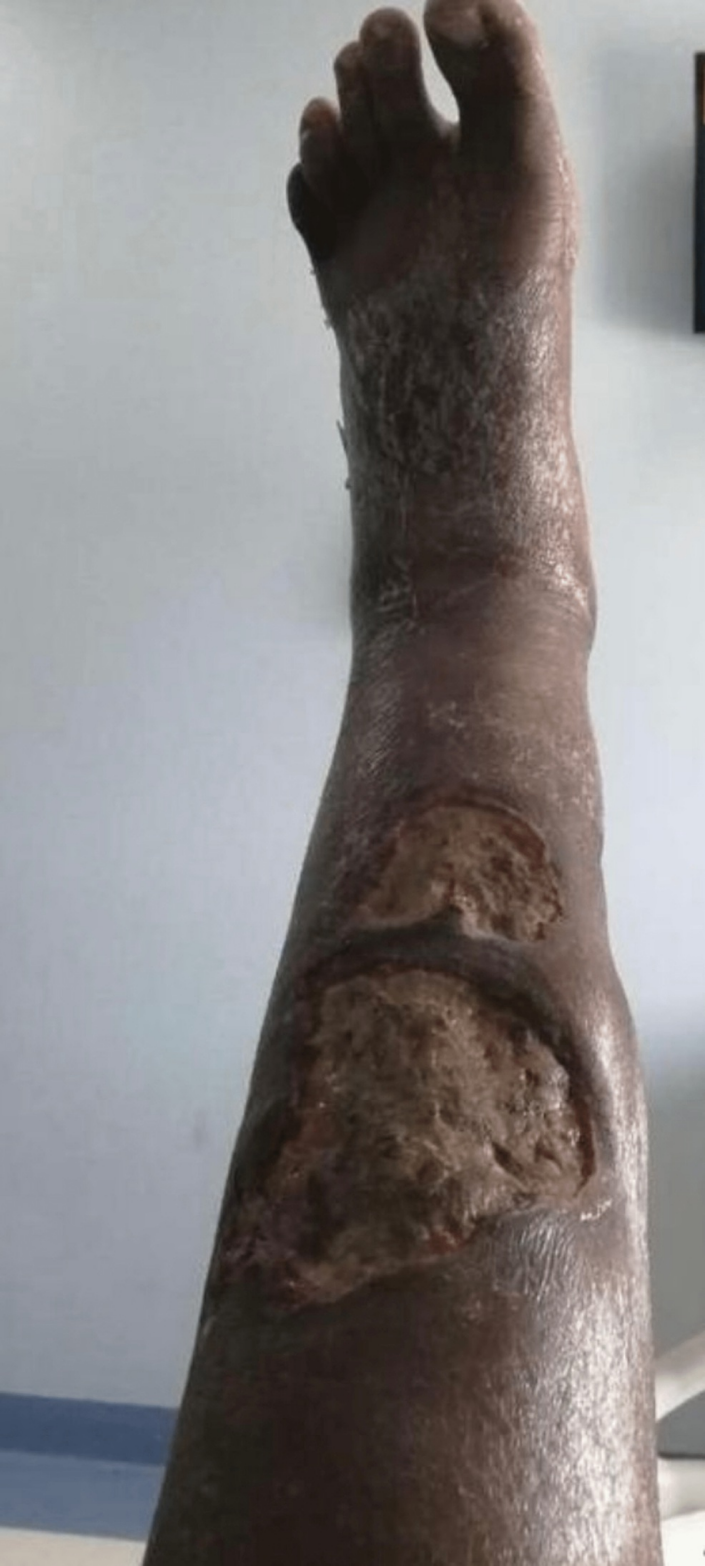
The primary lower leg ulcers of the patient

A 27-year-old male patient, medically free, with a positive surgical history for gastric bypass surgery as well as laparoscopic gastrectomy three years back, presented to our institute with an eight-day history of generalized edema, fatigue, dizziness, decreased urine output in addition to left leg nonhealing ulcers. The patient had been complaining of a leg ulcer for around six months, which had failed to be treated by various topical medications, including topical antibiotics and antimycotic creams that have been prescribed by general practitioners. The patient is a non-smoker and a non-alcoholic. There was no family history of such a complaint, as well as no family history of ulcerative colitis or Crohn's disease. Upon physical examination, the patient was afebrile, tachycardic, and tachypneic, with bilateral pitting lower limb edema, which extended up to the lower abdomen. While evaluating blood supply, a distal pulse could not be appreciated, with multiple large ulcers in the lower part of the left leg and foot (Figure 1).

Laboratory investigations showed random blood glucose: 5 mmol/L (normal range 5.5-7.7), serum potassium: 3.66 mmol/L (normal range is 3.4-5.1), lactic dehydrogenase: 319 U/L (normal range is 100-200), white blood cells: 13.91 (normal range is 4-10), hemoglobin: 7.8 g/dL (normal range is 13-17), and P folate serum: 2.98 ng/mL (normal range is 3-18).

The patient was admitted to the surgical ward and found to be malnourished with multiple organ failure. A biopsy of the non-healing ulcer was taken, with a histopathological result showing diffuse necroinflammatory and suppurative “neutrophilic” inflammation confirming pyoderma gangrenosum. A CT scan of the abdomen and pelvis with oral contrast demonstrated no extraluminal contrast leak, mild diffuse pancreatic atrophy, and diffuse gallbladder wall thickening with no pericholecystic fat stranding or fluid.

**Figure 2:**
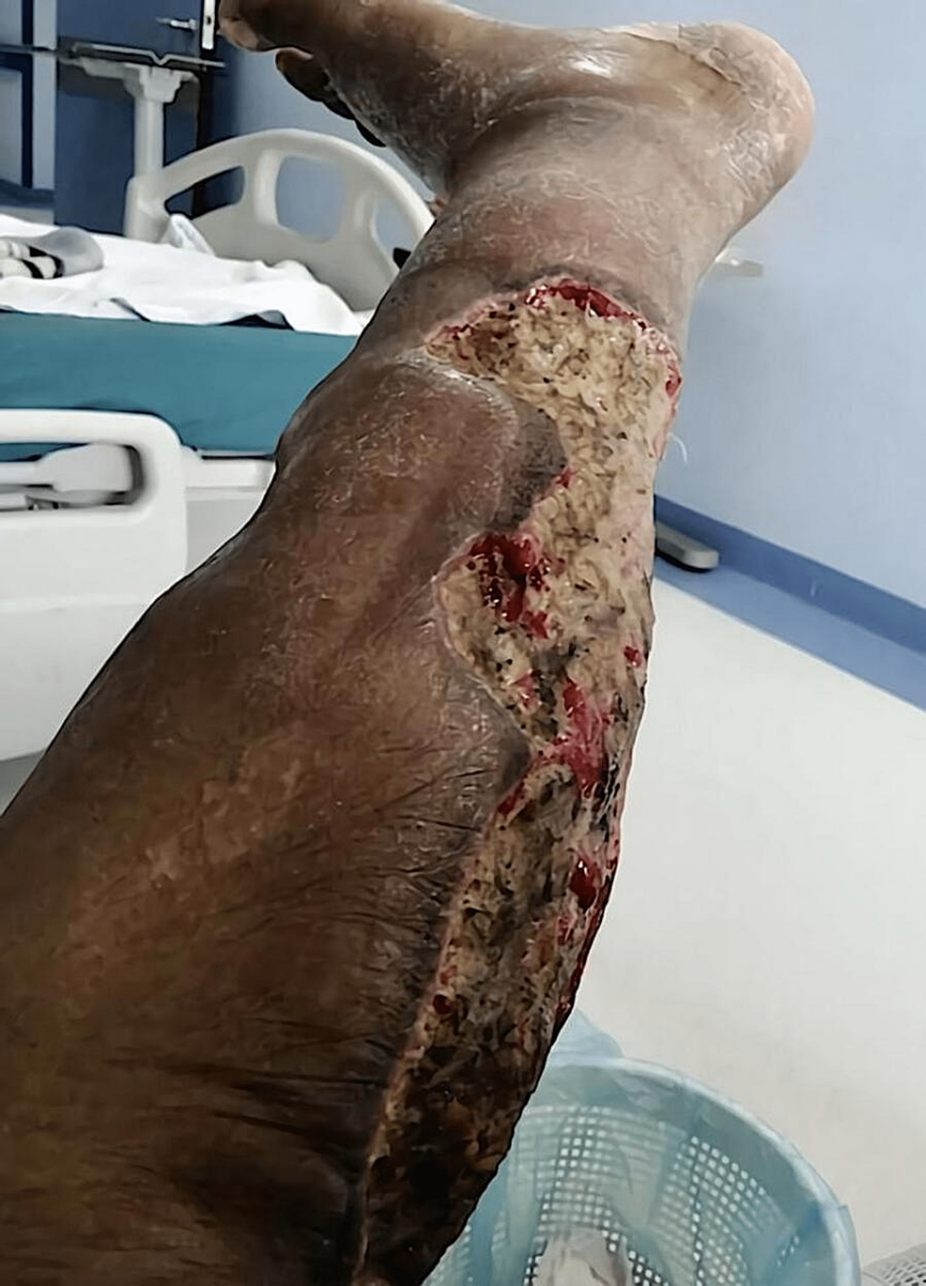
Post-surgical debridement

The patient was managed with surgical debridement (Figure 2), vacuum-assisted closure, and dressing with silver-cell and intravenous (IV) antibiotics.

**Figure 3:**
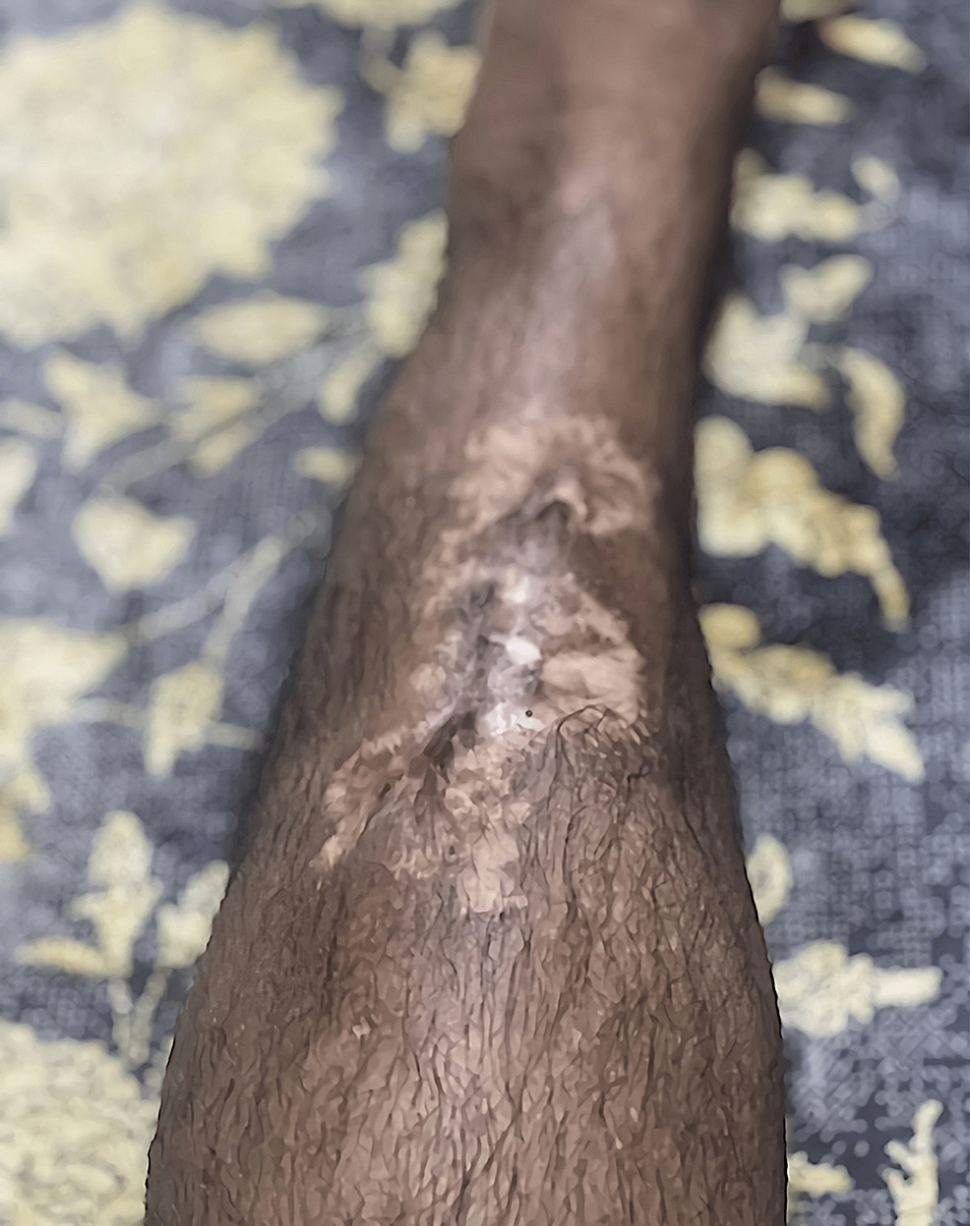
Healed ulcers after the treatment

**Figure 4:**
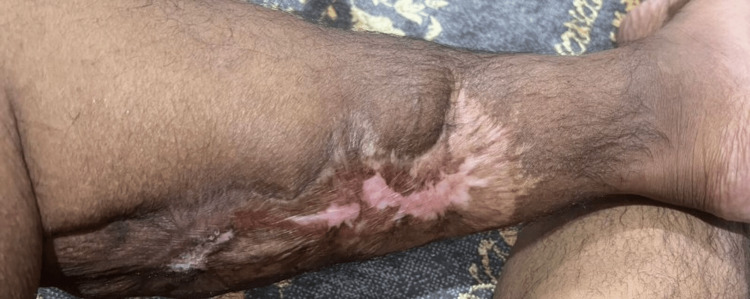
Healed ulcers after the treatment

As the patient was malnourished, he was managed with IV supplements initially and then shifted to oral. The patient has improved dramatically and was instructed to follow up in the outpatient clinic. Upon discharge, the patient was given vitamin B complex, vitamin D 5000 IU, zinc sulfate 30 mg orally (PO) once daily, folic acid 5 mg PO, infliximab 200 mg IV injections every two weeks for six weeks, and vitamin B12 IM weekly for three consecutive doses. The leg ulcer was improved with a satisfactory result (Figures 3, 4).

## Discussion

Pyoderma gangrenosum (PG) is defined as an inflammatory condition of the skin characterized histologically by necrosis, vasculitis, and ulceration and was first described almost 100 years ago. However, there are no known identifiable causes, nor uniformly accepted diagnostic criteria [5]. PG can be classified into four categories that are currently widely accepted: classic (ulcerative), bullous, pustular, and vegetative [4]. It is thought to affect patients between the ages of 25 and 54 without any gender preferences, with an estimated global incidence of three to 10 cases per million people per year. Moreover, children were involved in only 4% of the cases [4,7-8]. PG can manifest itself anywhere on the body. However, the lower legs (peritibia) are most typically affected when it comes to predilection sites in adults. That being said, in children, PG typically involves the lower extremities, buttocks, and perianal region, as well as the head and neck. Little trauma was reported to precipitate the lesion in almost one-third of the patients with PG, a phenomenon that’s known as "pethargy" [4,9].

Although PG is frequently associated with different systemic and inflammatory diseases such as inflammatory bowel diseases, rheumatologic diseases, or hematological malignancy [10], our case has demonstrated the possibility of having a young medically free patient with PG with the prospect of early diagnosis and satisfactory results. On many occasions, PG is characteristically seen as a reactive, non-infectious inflammatory dermatosis with neutrophil-predominant infiltrates [11], which has been also seen in the histopathology of our case. It is also noticeable that our case has diffuse gallbladder wall thickening, which could pave the way to cholecystitis. This finding is consistent with the Bittencourt et al. article that reported a case where PG has been preceded by cholecystitis [12].

Avoidance of triggers, good wound care, adequate pain management, and topical, systemic, and targeted immunomodulatory treatments may all be part of the best course of treatment for PG [6]. Although local management may be satisfactory in mild cases, in severe and extensive cases, systemic immunosuppressants are frequently the mainstay treatment [13]. In our patient, the extent of the injury, as well as the late presentation, prompted the use of surgical debridement, as it is commonly needed when there is a necessity for reconstruction [14]. As there is still no clear gold standard or even international guideline to handle PG cases yet [15-16], we believe that combining both medical and surgical pathways would result in faster, better, and more satisfactory healing as seen in our patient.

There is mostly only one published article describing the appearance of PG following gastric bypass surgery [17]. Bowel-associated dermatitis arthritis syndrome, also known as bowel bypass syndrome, is a well-known complication of a jejunoileal bypass as well as biliopancreatic diversion [18,19]. Recurrent fever, polyarthralgia/polyarthritis, myalgia, tenosynovitis, and protean cutaneous manifestations are the most recognized manifestations of this syndrome [20]. Patients affected by bowel bypass syndrome always have skin lesions that are described as PG. However, those skin lesions could mimic the features of other neutrophilic dermatoses such as Sweet’s syndrome [17]. The deposition of circulating immune complexes containing bacterial antigens in relation to bacterial overgrowth in the bowel is suggested to be the cause of bowel bypass syndrome. In fact, the actual reason for such a syndrome is not yet known [21]. Although our patient hasn't complained of arthritis symptoms, which is an important feature of bowel-associated dermatitis arthritis syndrome as well as tenosynovitis, we think that his skin lesions and ulcers are highly related to his previous bowel bypass surgery.

## Conclusions

Pyoderma gangrenosum is a rare disease, and the clinical presentation varies from one patient to another. Since there is still no gold standard treatment for the disease, the management plan selection is complicated. In our case, we avoid complicated reconstruction treatment and skin grafting with a good ulcer healing result. Clinical follow-up for every patient diagnosed with pyoderma gangrenosum post-treatment is crucial in order to assess the healing process and avoid any further complications.
